# World Trade Center Exposure and Posttraumatic Growth: Assessing Positive Psychological Change 15 Years after 9/11

**DOI:** 10.3390/ijerph18010104

**Published:** 2020-12-25

**Authors:** Cristina D. Pollari, Jennifer Brite, Robert M. Brackbill, Lisa M. Gargano, Shane W. Adams, Pninit Russo-Netzer, Jonathan Davidov, Victoria Banyard, James E. Cone

**Affiliations:** 1 Division of Epidemiology, New York City Department of Health and Mental Hygiene, World Trade Center Health Registry, Long Island City, NY 11101, USA; jbrite@health.nyc.gov (J.B.); rbrackbi@health.nyc.gov (R.M.B.); lisa.gargano@gmail.com (L.M.G.); jcone@health.nyc.gov (J.E.C.); 2 Department of Psychology, John Jay College of Criminal Justice, City University of New York, New York, NY 10019, USA; shadams@jjay.cuny.edu; 3 Department of Psychology, The Graduate Center, City University of New York, New York, NY 10016, USA; 4 Department of Counseling and Human Development, University of Haifa, Haifa 3498838, Israel; pninit.russonetzer@gmail.com (P.R.-N.); jdavidov74@gmail.com (J.D.); 5 School of Advanced Studies, Achva Academic College, Arugot 7980400, Israel; 6 School of Social Work, Rutgers University, New Brunswick, NJ 08901, USA; vb358@ssw.rutgers.edu

**Keywords:** disaster, World Trade Center, 9/11, posttraumatic growth, well-being

## Abstract

We evaluated the presence of posttraumatic growth (PTG) among survivors of the 9/11 terrorist attack and how indicators of psychosocial well-being, direct 9/11-related exposure, and posttraumatic stress symptoms (PTSS) relate to PTG. PTG was examined among 4934 participants using the Posttraumatic Growth Inventory (PTGI). A confirmatory factor analysis (CFA) was conducted to determine if the original factor structure of the PTGI fits our data and principal component analysis (PCA) to identify the appropriate factor structure. Multivariable linear regression models were used to examine the association between PTG and indicators of psychosocial well-being, 9/11-related exposure, and PTSS, controlling for covariates. CFA identified a two-factor structure of the PTGI as a better fit than the original five-factor model. Participants who experienced very high 9/11-related exposure level (ß = 7.72; 95% CI: 5.75–9.70), higher PTSS at waves 1 (ß = 0.13; 95% CI: 0.08–0.18) and 2 (ß = 0.09; 95% CI: 0.05–0.14), high social integration (ß = 5.71; 95% CI: 4.47, 6.96), greater social support (ß = 0.49; 95% CI: 0.37, 0.61), and higher self-efficacy (ß = 1.26; 95% CI: 1.04, 1.48) had higher PTGI scores. Our findings suggest PTG is present, 15 years following the 9/11 terrorist attack. Very high-level 9/11 exposure, PTSS, and indicators of psychosocial well-being were associated with PTG.

## 1. Introduction

Negative health outcomes are highly emphasized in public health and related disciplines. While these outcomes are important and necessary to monitor, positive outcomes and attributes of well-being are an integral aspect of one’s health [1]. The negative physical and mental health effects of the World Trade Center (WTC) disaster have been well documented [2,3,4,5,6,7]. Despite there being an overwhelming amount of evidence showing the negative effects of trauma, recent research has explored the positive changes that can arise [8,9,10,11]. The concept of posttraumatic growth (PTG), developed by Tedeschi and Calhoun, refers to the positive psychological change following a traumatic event that results in higher-level functioning [12]. Unlike resilience, PTG allows individuals to surpass pre-trauma functioning and not simply return to their baseline level of functioning [13]. The exact mechanisms of PTG remain unknown; Tedeschi and Calhoun suggest that PTG is not a direct result of trauma. Based on prior research, they build on cognitive schemas and how individuals interpret their experiences through a set of core beliefs, perceptions and understanding of the world [13]. Once a traumatic event is experienced, the cognitive schema is disrupted or challenged and the individual struggles with their new reality; it is through this process that PTG emerges [13].

In order to quantify the experience of growth, the Posttraumatic Growth Inventory (PTGI) was developed, measuring five domains of PTG. The five domains of PTG include changes in relating to others, new possibilities, personal strength, spiritual change, and appreciation for life [12]. However, some studies have identified other factor structures in different trauma survivors, [9,14,15,16,17,18], ranging from one to five domains. Even with these findings, it is still unclear whether PTG should be represented as a unitary construct or composed of multiple higher-order factors [19].

PTG has been found to exist in various populations after facing a traumatic event, including victims of the Oslo and 9/11 terror attacks [10,11,20] and mass violence [21], those with physical illnesses [9,21,22,23,24], sexual abuse victims [8], college students experiencing trauma [14,15,25], and veterans [26]. A higher level of PTG is generally associated with greater trauma and those who struggle with trauma are more likely to experience PTG [13]. PTG also appears to be time-sensitive; emerging shortly after a traumatic event, and has also been shown to increase with time [23]. Interestingly, PTG was associated with higher levels of post-traumatic stress disorder (PTSD) [10] and in some studies was a correlate of well-being [27,28,29], while in others it seemed precipitated by higher levels of distress [30]. Due to these conflicting findings in the literature, more research is needed to examine the relationship between PTG and psychological distress (PTSD) and psychological well-being (self-efficacy, social integration, and social support).

The relationship between PTG and direct 11 September 2001 (9/11) terror attack-related exposures has yet to be studied. Previous studies either evaluated indirect 9/11 exposure through television [11,20] or assessed personality and mental health factors associated with PTG in a 9/11 population but did not examine 9/11 exposures directly [31]. These prior studies measured PTG within one year of the 9/11 attack and to our knowledge, no study has evaluated PTG several years after 9/11. While an abundance of research has examined maladaptive responses to direct 9/11 exposure and related psychopathology, very few studies have examined positive adaptations, including the potential for PTG. This paper will help evaluate the validity of PTG, as a construct, in a large sample of individuals exposed to mass trauma. In addition, it will examine key psychosocial correlates and increase knowledge regarding potential factors that may contribute to PTG. The aims of this study were to (1) assess the pattern of subscales for the PTGI among 9/11 survivors; (2) evaluate whether direct 9/11-related exposure, indicators of psychosocial well-being, and posttraumatic stress symptoms (PTSS) are associated with PTG, 15 years after 9/11.

## 2. Materials and Methods

### 2.1. Participants

The World Trade Center Health Registry (WTCHR) was created by the New York City Department of Health and Mental Hygiene to monitor the physical and mental health effects of the 9/11 terror attack. The WTCHR is a cohort study that includes 71,426 participants. The participants comprise rescue and recovery workers, Lower Manhattan residents, local workers, school students and staff, and occupants/passersby on 9/11. Data were collected over 4 survey waves: Wave 1 (2003–2004), Wave 2 (2006–2007), Wave 3 (2011–2012), and Wave 4 (2015–2016). Details of recruitment and data collection are published elsewhere [2,4].

The current analysis included 4934 participants from an in-depth study on injury, Health and Quality of Life (HQoL) 15 years after 9/11 (N = 6544). To be eligible for the HQoL, participants had to have completed all four survey waves, be ≥18 years of age, and speak English. We excluded participants (N = 1610) who did not have complete data on the exposures, outcome, and covariates. There were not any significant differences between participants who were included and excluded from the study. The WTCHR protocol was approved by the Institutional Review Boards at the Centers for Disease Control and Prevention and the NYC Department of Health and Mental Hygiene. Informed consent was obtained from participants at enrollment into the WTCHR.

### 2.2. Outcome

Posttraumatic growth was assessed in the HQoL, using the PTGI [12], a self-reported 21-item measure assessing positive change experienced after trauma. The PTGI uses a 6-point Likert scale from 0 (“I did not experience this change as a result of my crisis”) to 5 (“I experienced this change to a very great degree as a result of my crisis”). Sample items include: “I have a greater appreciation for the value of my own life” and “I have more compassion for others.” PTGI item 7, “I established a new path for my life”, was inadvertently omitted and was not administered to any participants. Item 7 was considered missing at random and all analyses were done excluding this item. Our PTGI total score included the sum of the 20 items. Scores ranged from 0 to 100, with a higher score indicating greater growth. Moderate-to-high posttraumatic growth was defined as having an average of all scores across the 20-item questionnaire as ≥3 [32,33].

### 2.3. Exposures

PTSS was assessed across all four survey waves using the PTSD Checklist (PCL 17-item) [34,35,36] questionnaire. The PCL is a self-reported measure, which includes items focused on re-experiencing symptoms specific to 9/11 in the past 30 days, with responses ranging from 1 (not at all) to 5 (extremely). PCL total score was calculated by summing all 17 items, for each survey wave. PCL improvement was characterized by the change in PCL total score from Wave 1 to Wave 4. PCL improvement was categorized as no positive change (<5 points), minimal positive change (5–9 points), moderate positive change (10–19 points), or significant positive change (≥20 points) [37].

The 9/11 exposure scale is a composite score involving 12 questions capturing various levels of traumatic experiences [38]. The 9/11 exposure experiences included: being in the North or South WTC towers at the time of the attack; witnessing three or more events (seeing planes hit the buildings, people fall or jump from buildings, people injured, or people running); fear of being injured or killed; having a relative killed on 9/11; having a friend killed on 9/11; having a co-worker killed on 9/11; experiencing an intense dust cloud; losing possessions; sustaining injury more serious than eye irritation/injury; being a rescue/recovery or clean-up worker; having evacuated one’s home for at least 48 h after 9/11; having lost one’s job because of 9/11. The 12 items were summed and then categorized as none/low (0–1 exposures), medium (2–3), high (4–5), and very high (≥6) [38].

Information on social integration was collected at Wave 2, social support at Wave 3, and self-efficacy at Wave 4. Social integration was based on the RAND Social Health Battery [39], which included the following items: having 1 or more close friends, having visited/talked to/emailed friends at least twice in the last 30 days, attended a religious service at least twice in last 30 days, or been actively involved in a volunteer organization or club in last 30 days. The 4 items were summed and categorized as low/medium (0–2) and high (≥3). Social support was assessed by the Social Support Survey for the Medical Outcomes Study, 5-item version [40]. Questions included how often someone is available to: have a good time with, hug you, take you to the doctor, prepare your meals if you are unable, and understand your problems. The five items were summed with scores ranging from 0 to 20, with a higher score indicating greater social support. Information on perceived self-efficacy was based on five items from the General Self-Efficacy Scale (GSE) [41]. Participants were asked how well they can handle unforeseen problems. The items were “It is easy for me to stick to my aims and accomplish my goals”, “I am confident that I could deal efficiently with unexpected events”, “Thanks to my resourcefulness, I know how to handle unforeseen situations”, “I can remain calm when facing difficulties because I can rely on my coping abilities”, and “No matter what comes my way, I am usually able to handle it”. Items were rated on a four-point scale (0 = not at all true to 4 = exactly true) and were summed to create a total score ranging from 0 to 20, with a higher score reflecting greater self-efficacy.

### 2.4. Covariates

Demographic data were collected at Wave 1. They included age at 9/11 (years), sex (male or female), race/ethnicity (White Non-Hispanic, Black Non-Hispanic, Hispanic/Latino, Asian, or Multiracial/other), education (less than high school, high school/GED, some college, or college/postgraduate), and marital status (married/living with partner, divorced/separated, widowed, or never married).

### 2.5. Statistical Analysis

We conducted confirmatory factor analysis (CFA) to determine if the original factor structure of the PTGI fit the WTCHR data. Model fit was assessed using the chi-square statistic (χ^2^), comparative fit index (CFI), the root mean square error of approximation (RMSEA), and the Tucker–Lewis index (TLI). To determine a good fit, we used cutoff values close to 0.06 for RMSEA, ≥0.95 for CFI and TLI, and non-significant χ^2^ [42]. Principal component analysis (PCA) [43] with a varimax rotation was used to identify the appropriate factor structure for our data. PCA accounts for all the variability among the variables and the varimax rotation aids in creating clearer factor loadings. We chose factors with eigenvalues >1 [12]. To be consistent with other studies [12,17,44], we assigned an item to a factor if it loaded greater than 0.5 and less than 0.4 on any other factor.

Overall frequencies of demographic characteristics, indicators of well-being, PTSS, and 9/11-related exposure were performed. Bivariate analysis was performed for each demographic characteristic, indicators of well-being, PTSS, and 9/11-related exposure with moderate-to-high PTG, using chi-squared tests for categorical variables and Kruskal–Wallis for non-normal continuous variables. Multivariable linear regression was used to assess 9/11 exposure, PTSS, and indicators of well-being with PTG. We ran 9 separate models, one model for each exposure, controlling for age, race\ethnicity, sex, education, and marital status. Betas, 95% confidence intervals, and *p*-values were computed. The significance level was set at a 2-sided value of alpha <0.05. A sensitivity analysis was conducted to determine if the factor structure identified in our sample would produce similar results as the 20 items in the bivariate and multivariable regression models.

All analyses were conducted using SAS 9.4 (SAS Institute, Inc., Cary, NC, USA).

## 3. Results

The original factor structure of the PTGI did not apply to the WTCHR enrollees. CFA results showed that this model did not fit our data well, χ^2^ = 19,753.8, *p* < 0.0001, CFI = 0.758, TLI = 0.721, and RMSEA = 0.155. As the original five-factor model was not a good fit, principal component analysis (PCA) with varimax rotation was done on the 20 items of the PTGI. Two factors were identified, with eigenvalues greater than one and accounted for 64.3% of the total variance (Table 1). Factor 1, labeled interconnectedness, accounted for 58.9% of the variance and included six items. The highest factor loadings came from the original “relating to others” factor (items 6, 8, 20, and 21), one loading from “new possibilities” (item 14), and another from “spiritual change” (item18). Factor 2, labeled personal growth, accounted for 5.4% of the variance. The highest loadings for factor 2 came from the original “appreciation of life” factor (items 1 and 2) and one factor from “new possibilities” (item 3) and another from “personal strength” (item 4). The two-factor structure of the PTGI was a better fit than the original five-factor model (χ^2^ = 1646.4, *p* < 0.0001, CFI = 0.941, TLI = 0.922, and RMSEA = 0.098).

The mean PTGI score was 48.18 (standard deviation = 22.94), with 34.27% having moderate-to-high PTG (Not shown in tables). Table 2 shows the frequencies of demographic characteristics, indicators of well-being, PTSS, and 9/11-related exposure for the overall sample and by moderate-to-high PTG. The overall median age at 9/11 was 43 years old and the majority of participants were male (65%), White Non-Hispanic (79%), had at least a Bachelor’s degree (58%), and married/living with a partner (70%). The median score for self-efficacy was 16, the social support median was 15, and most participants experienced low/medium level of social integration (51%). The median PCL total scores for waves 1–4 were 26, 28, 27, and 25, respectively.

Those with moderate-to-high PTG were more likely to be slightly older, female, Non-White, slightly less educated, have greater levels of self-efficacy, higher social support, higher social integration, higher PCL total score at W1, and higher 9/11-related exposure when compared to those without moderate-to-high PTG (Table 2).

Table 3 shows the multivariable linear regression analysis of 9/11-related exposures, PTSS, and indicators of well-being with PTG. PCL improvement and 9/11-related exposure both showed a dose–response relationship with PTG. Those with very high 9/11-related exposure had a 7.72-point increase in the PTGI (95% CI: 5.75–9.70). PCL improvement of ≥20 points was associated with an 8.84–point increase in PTGI (95% CI: 5.74–11.94). A point increase in PCL total scores at Wave 1 (ß = 0.13, 95% CI: 0.08–0.18) and Wave 2 (ß = 0.09, 95% CI: 0.05–0.14) showed a slight significant increase in the PTGI total score. A point increase in social support or self-efficacy showed a slight increase in PTGI.

The sensitivity analysis with the 10 items identified from the two-factor structure showed very similar findings as the 20 items (Table S1). Participants who experienced higher 9/11-related exposure, higher PCL total scores at waves 1 and 2, high social integration, greater social support, and higher self-efficacy showed an increase in PTGI total score (Table S2).

## 4. Discussion

The aim of the study was to evaluate the presence of PTG among 9/11 survivors and explore how demographic factors, indicators of psychosocial well-being, direct 9/11-related exposure, and PTSS relate to PTG. We found that PTG is present among 9/11 survivors, 15 years following the 9/11 terrorist attack. The demographic factors associated with moderate-to-high PTG were being female, a racial minority, and having less education. Racial minorities are known to face greater historical, economical, and social barriers [45]. The cumulative effect of pre-existing challenges with 9/11-related exposure contribute to a greater lifetime experience of trauma exposure. These additional challenges in one’s life and its increased association for PTG, supports the theory that PTG manifests with more struggle [13]. Women experiencing more PTG than men is also consistent with other studies [12,46]. Previous research has identified gender roles and not gender itself to be associated with PTG, specifically, female traits, such as empathy and openness [47]. Our findings indicate social support, social integration, and self-efficacy are valuable resources in the development of PTG. Our results are consistent with other studies that have reported social support predicts greater PTG [28,29].

The original five-factor structure did not fit our data and a two-factor design showed a better fit. Levine et al. [48] also identified a two-factor structure, which indicates PTG may be a broader construct than what other studies [12,17,18,25] have found. The number of PTG factors and the domains identified may depend on the study design, sample, and the salience of these domains to the population of interest [49]. Findings suggest that the domains of interconnectedness and personal growth may be particularly salient to 9/11 survivors and the unique difficulties faced in this community following exposure to mass trauma. Within the current factor structure, social support and integration as well as new priorities and a greater appreciation of life were viewed as central to retrospective perceptions of personal growth 15 years after 9/11. In speculation, due to the largely shared-experience of 9/11, in which people were forced to rely on others to either evacuate the WTC towers, navigate the dust-covered streets of lower Manhattan, or work together to recover survivors\clear debris, in addition to living in a community that had to live with constant physical and psychological reminders of the event, led to increased reliance on others to cope effectively. This finding is consistent with recent research [50] that indicates marked psychological improvements following adversity are associated with the perception of psychosocial gains and largely unrelated to ruminative processing of the event or benefit finding.

The identification of interconnectedness and the perception of personal growth as dominant factors may also be a product of current methodology in which participants were asked to reflect on a period of 15 years and identify a few specific personal changes. When reflecting on their own positive adaptations to adversity years later, participants’ perceptions of personal growth may have become broadly associated with more observable interpersonal relationships and inherent values of interconnectedness rather than less observable and more intrinsic motivators. The idea that experiencing a traumatic event creates a sense of collective history, or even a reference point to understanding oneself may be developed further to highlight the contribution of the present findings to existing literature. From a random group of people exposed to a traumatic event, they can be viewed as a potential resource which may stimulate PTG, based on shared experience, solidarity, social bonding, a sense of belongingness and interconnectedness between group members. This resonates with previous research, showing that a sense of belongingness to a community may provide a potential “protective shield” against a reality of terror and war [51].

Increased 9/11-related exposure was significantly associated with PTG. Our findings are consistent with other studies showing that posttraumatic growth can occur after some length of time following a traumatic event [23]. We also found that PTG is associated with PTSS reductions over time. PTSD and PTG have been studied and their relationship has shown mixed results, with results indicating a positive [30,52] or curvilinear [48] relationship. A curvilinear relationship has shown the highest levels of PTG are associated with moderate levels of PTSD [48]. However, the current study did not indicate a strong linear or nonlinear relationship. In our study, PTG was associated with PTSS in the immediate years after the 9/11 attack, rather than several years later. Findings indicate that the relationship between PTG and PTSS may be temporary as the two are positively associated until a certain time point.

A major strength of this study was the longitudinal design which allowed us to establish temporal order of the relationship between 9/11-related exposure, PTSS, and PTG. The large sample size ensured adequate power. The administration of the PTGI and measures of well-being provided multidimensional assessments of positive adjustments and growth following 9/11, which help to strengthen evidence of PTG [53]. However, the current study had a few limitations. Our sample consisted of everyone who was directly exposed to 9/11 and did not have a control group as a comparison. Although, we had a longitudinal cohort, PTG was only assessed at one point in time, and we were unable to explore PTG at various time points after the 9/11 attack. Item 7 from the PTGI was inadvertently omitted from our questionnaire, which makes it difficult to accurately compare our factor structure to similar studies.

The evaluation of PTG and distress over time among terror attack survivors should be further explored. Our study and many others have focused on PTG among adults or adolescents. More work is needed to explore how PTG manifests among children exposed to trauma, especially terror attacks, since these events impact all individuals in a community. Psychological well-being is not only a desired outcome but also impacts physical health [1]. Future research should also examine how PTG impacts physical health and mortality. Trauma treatment for 9/11 victims should consider PTG and the extent it can aid patients facing adversity and how interconnectedness and personal growth play a role.

## 5. Conclusions

The 9/11 terrorist attack exposed thousands of people in New York City to trauma. While most 9/11 research emphasizes the negative impacts of trauma exposure, our study highlights the importance of exploring positive outcomes. This study used data up to 15 years post-9/11 to identify the presence of PTG. PTSS was associated with PTG in the immediate years after the 9/11, rather than several years later. A very high level of 9/11-related exposure and greater levels of social integration, social support, and self-efficacy showed a slight increase in PTG.

## Figures and Tables

**Table 1:** Factor Loadings of 20 Posttraumatic Growth Inventory (PTGI) items.

PTGI Item and Factor	Factor in Original PTGI	F1	F2
Factor I: Interconnectedness (58.9% of variance)			
6. I more clearly see that I can count on people in times of trouble.	I	0.78	
8. I have a greater sense of closeness with others.	I	0.78	0.34
14. New opportunities are available which would not have been otherwise.	II	0.53	0.39
18. I have a stronger religious faith.	IV	0.63	
20. I learned a great deal about how wonderful people are.	I	0.80	
21. I better accept needing others.	I	0.81	
Factor II: Personal growth (5.4% of variance)			
1. I changed my priorities about what is important in life.	V		0.79
2. I have a greater appreciation for the value of my own life.	V	0.36	0.73
3. I developed new interests.	II	0.32	0.69
4. I have a greater feeling of self-reliance.	III	0.39	0.70
Items that failed to load			
5. I have a better understanding of spiritual matters.	IV	0.60	0.42
9. I am more willing to express my emotions.	I	0.68	0.41
10. I know better that I can handle difficulties.	III	0.51	0.61
11. I am able to do better things with my life.	II	0.60	0.63
12. I am better able to accept the way things work out.	III	0.57	0.58
13. I can better appreciate each day.	V	0.56	0.63
15. I have more compassion for others.	I	0.62	0.50
16. I put more effort into my relationships.	I	0.63	0.55
17. I am more likely to try to change things which need changing.	II	0.58	0.59
19. I discovered that I’m stronger than I thought I was.	III	0.58	0.54

Note: I = Relating to Others; II = New Possibilities; III= Personal Strength; IV = Spiritual Change; V = Appreciation of Life. Only factor loadings >0.3 are shown. PTGI item 7, “I established a new path for my life”, is not included.

**Table 2:** Characteristics of Study Sample by Moderate-to-high Posttraumatic Growth.

	Moderate-to-High Posttraumatic Growth			
	Overall	Yes	No	*p*-Value
	(N = 4934)	(N = 1691)	(N = 3243)	
**Demographics**				
Age at 9/11 (year)	43.0 (35.0, 49.0)	44.0 (37.0, 51.0)	42.0 (34.0, 49.0)	<0.001
Gender				<0.001
Male	3227 (65%)	1017 (60%)	2210 (68%)	
Female	1707 (35%)	674 (40%)	1033 (32%)	
Race/Ethnicity				<0.001
White Non-Hispanic	3922 (79%)	1207 (71%)	2715 (84%)	
Black or African American	322 (7%)	201 (12%)	121 (4%)	
Hispanic or Latino (any race)	407 (8%)	197 (12%)	210 (6%)	
Asian (includes Native Hawaiian/Pacific) Islander	161 (3%)	52 (3%)	109 (3%)	
Multiracial/Other	122 (2%)	34 (2%)	88 (3%)	
Education				<0.001
Less than high school	41 (1%)	11 (1%)	30 (1%)	
High school only	761 (15%)	306 (18%)	455 (14%)	
Some college	1272 (26%)	522 (31%)	750 (23%)	
At least a Bachelor’s	2860 (58%)	852 (50%)	2008 (62%)	
Marital Status				0.09
Married or living with partner	3449 (70%)	1177 (70%)	2272 (70%)	
Divorced or separated	476 (10%)	171 (10%)	305 (9%)	
Widowed	65 (1%)	31 (2%)	34 (1%)	
Never married	944 (19%)	312 (18%)	632 (19%)	
Income				<0.001
Less than USD 50,000	852 (19%)	335 (22%)	517 (17%)	
USD 50,000+	3665 (81%)	1214 (78%)	2451 (83%)	
**Indicators of psychosocial well-being**				
Self-Efficacy	16.0 (15.0, 19.0)	17.0 (15.0, 20.0)	16.0 (15.0, 19.0)	<0.001
Social Support W3	15.0 (11.0, 20.0)	16.0 (12.0, 20.0)	15.0 (11.0, 19.0)	<0.001
Social Integration W2				<0.001
Low/medium	2497 (51%)	707 (42%)	1790 (55%)	
High	2437 (49%)	984 (58%)	1453 (45%)	
**PTSS**				
PTSD W1	26.0 (20.0, 36.0)	27.0 (21.0, 38.0)	25.0 (20.0, 36.0)	<0.001
PTSD W2	28.0 (21.0, 40.0)	28.0 (21.0, 40.0)	27.0 (21.0, 39.0)	0.21
PTSD W3	27.0 (20.0, 38.0)	27.0 (20.0, 38.0)	27.0 (20.0, 38.0)	0.62
PTSD W4	25.0 (19.0, 35.0)	24.0 (19.0, 34.0)	25.0 (19.0, 36.0)	0.04
**Direct 9/11-related Exposure**				
9/11 Exposure Scale				<0.001
None/Low	1057 (21%)	325 (19%)	732 (23%)	
Medium	1728 (35%)	557 (33%)	1171 (36%)	
High	1233 (25%)	450 (27%)	783 (24%)	
Very High	916 (19%)	359 (21%)	557 (17%)	

Note: Values expressed as N (%) or median (25th, 75th percentiles); *p*-value comparisons across groups for categorical variables are based on chi-square test of homogeneity; *p*-values for continuous variables are based on ANOVA or Kruskal-Wallis test for median. PTSS = post-traumatic stress symptoms; PTSD = post-traumatic stress disorder.

**Table 3:** Multivariable Linear Regression Models with 9/11 Exposure, PTSS, and Psychosocial Factors with Posttraumatic Growth.

Variable	B	95% CI	*p*
9/11 Exposure Scale *			
None/Low	Reference		
Medium	1.92	(0.23, 3.61)	0.0263
High	5.6	(3.78, 7.43)	<0.0001
Very High	7.72	(5.75, 9.70)	<0.0001
PCL Score W1 *	0.13	(0.08, 0.18)	<0.0001
PCL Score W2 *	0.09	(0.05, 0.14)	<0.0001
PCL Score W3 *	0.03	(−0.01, 0.08)	0.1392
PCL Score W4 *	−0.02	(−0.07, 0.03)	0.4139
PCL Improvement W1–W4 *			
<5 (No positive change)	Reference		
5 to 9 (Minimal positive change)	3.97	(2.17, 5.77)	<0.0001
10 to 19 (Moderate positive change)	5.31	(3.33, 7.29)	<0.0001
≥20 (Significant positive change)	8.84	(5.74, 11.94)	<0.0001
Social Integration *			
Low/medium	Reference		
High	5.71	(4.47, 6.96)	<0.0001
Social Support *	0.49	(0.37, 0.61)	<0.0001
Self-Efficacy *	1.26	(1.04, 1.48)	<0.0001

Note: * Adjusted for age, sex, race, education, and marital status; B = beta; 95% CI = 95% confidence interval; *p* = *p*-value.

## Data Availability

The data presented in this study are available on request from the World Trade Center Registry. The data are not publicly available due to privacy reasons.

## Supplementary Materials

The following are available online at https://www.mdpi.com/1660-4601/18/1/104/s1, Table S1: Characteristics of Study Sample by Moderate-to-high Posttraumatic Growth with 10 items; Table S2: Multivariable Linear Regression Models with 9/11 Related Exposures, PTSS, and Psychosocial Factors with Posttraumatic Growth (10-item total score).
