# Supplementary material for: World Trade Center Exposure and Posttraumatic Growth: Assessing Positive Psychological Change 15 Years after 9/11
Source: Int J Environ Res Public Health. 2020 Dec 25;18(1):104. doi: 10.3390/ijerph18010104 (PMC7795403; doi:10.3390/ijerph18010104)
Supplement: Supplementary file 1 [file ijerph-18-00104-s001.pdf]

Table S1. Characteristics of Study Sample by Moderate-to-high Posttraumatic Growth with 10 items.

		Moderate Posttraumatic Growth		
	Overall (N=4,934)	*Yes (N=1,522)*	*No (N=3,412)*	*p-value*
Characteristics				
	43.0 (35.0, 49.0)	44.0 (37.0, 51.0)	42.0 (34.0, 49.0)	<0.001
**Age (yr)**				
**Sex**				<0.001
Male	3,227 (65%)	917 (60%)	2,310 (68%)	
Female	1,707 (35%)	605 (40%)	1,102 (32%)	
**Race/Ethnicity**				<0.001
White	3,922 (79%)	1,092 (72%)	2,830 (83%)	
Black or African American	322 (7%)	180 (12%)	142 (4%)	
Hispanic or Latino (any race)	407 (8%)	174 (11%)	233 (7%)	
Asian (includes Native Hawaiian/Pacific Islander)	161 (3%)	47 (3%)	114 (3%)	
Multiracial/Other	122 (2%)	29 (2%)	93 (3%)	
**Education**				<0.001
Less than high school	41 (1%)	9 (1%)	32 (1%)	
High school only	761 (15%)	286 (19%)	475 (14%)	
Some college	1,272 (26%)	454 (30%)	818 (24%)	
At least a Bachelor's	2,860 (58%)	773 (51%)	2,087 (61%)	
**Marital Status**				0.11
Married or living with partner	3,449 (70%)	1,057 (69%)	2,392 (70%)	
Divorced or separated	476 (10%)	156 (10%)	320 (9%)	
Widowed	65 (1%)	28 (2%)	37 (1%)	
Never married	944 (19%)	281 (18%)	663 (19%)	
**Self-Efficacy**	16.0 (15.0, 19.0)	17.0 (15.0, 20.0)	16.0 (15.0, 19.0)	<0.001
**Social Support W3**	15.0 (11.0, 20.0)	16.0 (12.0, 20.0)	15.0 (11.0, 19.0)	<0.001
**Social Integration W2**				<0.001
Low/medium	2,497 (51%)	613 (40%)	1,884 (55%)	
High	2,437 (49%)	909 (60%)	1,528 (45%)	
**PCL Total Score W1**	26.0 (20.0, 36.0)	28.0 (21.0, 37.0)	25.0 (20.0, 36.0)	<0.001
**PCL Total Score W2**	28.0 (21.0, 40.0)	28.0 (21.0, 40.0)	27.0 (21.0, 39.0)	0.11
**PCL Total Score W3**	27.0 (20.0, 38.0)	27.0 (20.0, 38.0)	27.0 (20.0, 38.0)	0.42
**PCL Total Score W4**	25.0 (19.0, 35.0)	24.0 (19.0, 34.0)	25.0 (19.0, 36.0)	0.2
**9/11 Exposure**				<0.001
None/Low	1,057 (21%)	289 (19%)	768 (23%)	
Medium	1,728 (35%)	500 (33%)	1,228 (36%)	
High	1,233 (25%)	412 (27%)	821 (24%)	
Very High	916 (19%)	321 (21%)	595 (17%)	

Note: Values expressed as N (%) or median (25th, 75th percentiles). P-value comparisons across groups for categorical variables are based on chi-square test of homogeneity; p-values for continuous variables are based on ANOVA or Kruskal-Wallis test for median

Table S2. Multivariable Linear Regression Models with 9/11 Related Exposures, PTSS, and Psychosocial Factors with Posttraumatic Growth (10-item total score).

**Variable**	**ß**	**95% CI**	**p**
**9/11 Exposure***			
None/Low	Reference		
Medium	0.78	(-0.03, 1.60)	0.0603
High	2.67	(1.79, 3.55)	<0.0001
Very High	3.55	(2.60, 4.51)	<0.0001
**PCL Total Score W1***	0.06	(0.04, 0.09)	<0.0001
**PCL Total Score W2***	0.04	(0.02, 0.06)	<0.0001
**PCL Total Score W3***	0.02	(-0.00, 0.04)	0.0899
**PCL Total Score W4***	-0.01	(-0.03, 0.02)	0.6494

**PCL Improvement W1-W4***			
<5 (No positive change)	Reference		
5 to 9 (Minimal positive change)	1.75	(0.88, 2.61)	<0.0001
10 to 19 (Moderate positive change)	2.3	(1.34, 3.25)	<0.0001
≥ 20 (Significant positive change)	4.12	(2.63, 5.61)	<0.0001
**Social Integration ***			
Low/medium	Reference		
High	2.89	(2.30, 3.49)	<0.0001
**Social Support***	0.25	(0.19, 0.30)	<0.0001
**Self-Efficacy***	0.56	(0.46, 0.67)	<0.0001

*Adjusted for age, sex, race, education, and marital status

ß=beta; 95% CI= 95% Confidence Interval; p=p-value
